# Research on the Relative Placement Angle of the Induction Heater and the Channel in a Four-Channel Induction-Heating Tundish

**DOI:** 10.3390/ma17123011

**Published:** 2024-06-19

**Authors:** Xiqing Chen, Pu Wang, Hong Xiao, Siyan Lei, Haiyan Tang, Jiaquan Zhang

**Affiliations:** 1 School of Metallurgical and Ecological Engineering, University of Science and Technology Beijing, No. 30 Xueyuan Road, Haidian District, Beijing 100083, China; xiqing_ustb@163.com (X.C.);; 2 Magnetoelectric Research Institute, Hunan Zhongke Electric Co., Ltd., Yueyang 414000, China

**Keywords:** induction-heating tundish, four channel, numerical simulation, induction heater angle, electromagnetic field

## Abstract

In order to optimize the application effect of induction heating (IH) tundishes, a four-channel IH tundish is taken as the research object. Based on numerical simulation methods, the influence of different relative placement angles of induction heaters and channels on the electromagnetic field, flow field and temperature field of the tundish is investigated. We focus on comparing the magnetic flux density (B) and electromagnetic force (EMF) distribution of the channel. The results show that regardless of the relative placement angle between the heater and the channel, the distribution of B in the central circular cross-section of the channel is eccentric. When the heater rotates around channel 1 towards the bottom of the tundish, the distribution of B in the central circular cross-section of the channel changes from a horizontal eccentricity to a vertical one. Through the analysis of the B contour in the longitudinal section of the channel, the difference in effective magnetic flux density area (ΔA_B_) between the upper and lower parts of the channel can be obtained, thereby quantitatively analyzing the distribution of B in this section. The distribution pattern of ΔA_B_ is consistent with the distribution pattern of the electromagnetic force in the vertical direction (F_Z_) of the channel centerline. The ΔA_B_ and F_Z_ of channel 1 gradually increase as the heater rotates downwards, while those of channel 2 reach their maximum value at a rotation angle of 60°. Compared to the conventional placement, when the heater rotation angle is 60°, the outlet flow velocities at channel 1 and channel 2 decrease by 15% and 12%, respectively. However, the outlet temperature at channel 2 increases by 1.96 K, and the molten steel flow at the outlet of channel 1 and channel 2 no longer exhibits significant downward flow. This shows that when the heater rotation angle is 60°, it has a dual advantage. On the one hand, it is helpful to reduce the erosion of the molten steel on the channel and the bottom of the discharging chamber, and on the other hand, it can more effectively exert the heating effect of the induction heater on the molten steel in the channel. This presents a new approach to enhance the application effectiveness of IH tundish.

## 1. Introduction

With the development of society, the requirements for the grades and quality of steel products are becoming increasingly stringent [1,2]. The tundish, as an intermediate metallurgical container in the continuous-casting process, plays a crucial role in maintaining consistent steel superheat and achieving a steady temperature and casting speed. Reasonable flow behavior and stable temperature distribution within a tundish are key factors for controlling these parameters [3,4,5]. Tundish heating and molten steel temperature control are representative advanced technologies in the current continuous-casting production of special steel [6,7,8]. Many steel plants, especially those producing special steel, have begun to consider induction heating (IH) equipment for tundishes as essential equipment for their continuous-casting production lines. Similarly, in the past two decades, scholars have been gradually conducting in-depth theoretical research on IH tundishes [9,10].

Many scholars have focused their research on channel-type IH tundishes on changes in the electromagnetic field’s effects on the flow field, temperature field, and inclusion removal within a tundish after induction heating [11,12,13]. Wang et al. [14,15] used a combination of physical simulation and numerical simulation to conduct a detailed study of the electromagnetic field, flow field, temperature field, and inclusion removal behavior of the dual-channel tundish of a single-strand single-inductor. Research has shown that Joule heat is generated by induced current and that Joule heat is the highest on the surface of a channel. Electromagnetic force (EMF) is asymmetrically distributed in the channel and has a pinching effect on the molten steel in the channel. It is believed that molten steel is subject to thermal buoyancy and has an upward force. The flow trend evens out the temperature of the molten steel in the tundish, and increasing the heating power is more conducive to the removal of inclusions. Yue et al. [16,17] performed simulation calculations on a channel-type tundish with multi-strand double heaters and compared the differences in the flow of molten steel without induction heating, only Joule heat, and the combined action of Joule heat and EMF. It was revealed that in this structure, there may have been a phenomenon of molten steel scouring the bottom of the tundish in the region where the channel outlet and the discharging chamber were connected.

In order to optimize the effectiveness of induction-heating tundishes, scholars have made many efforts and attempts. Tang et al. [18] optimized the flow pattern of molten steel by adding flow control devices such as retaining walls and dams in the discharging chambers of multi-strand IH tundishes. This approach improved the consistency of each strand and reduced the dead ratio in multi-strand tundishes. Some scholars have also focused on optimizing the structure of the channels themselves. For example, Xing et al. [19,20] proposed curved heating channels, which not only enhance heating efficiency but also aid in the removal of inclusions. Some scholars have proposed new tundish structures, such as four-channel tundishes, butterfly channel tundishes, etc. In addition to optimizing the structure of tundishes, as mentioned above, scholars have conducted in-depth research on the parameters for IH equipment, thereby further optimizing the application effect of IH equipment. Yang et al. [21,22,23] proposed the use of intermittent heating throughout the entire casting process, which can reduce energy consumption while increasing the removal rate of inclusions. Wang et al. [24] studied coil placement methods, comparing the distribution of multi-physics fields in a multi-strand, dual-channel tundish when the coil was placed horizontally and vertically. It was pointed out that when the coil was placed horizontally, the molten steel flowing out of the channel could have an upward flow. Chen et al. [25] conducted a numerical simulation on the matching effects of tundishes with different channel numbers and single/double heaters. It was pointed out that for dual-channel tundishes, single or double heaters can be used with a same-direction current, while for four-channel tundishes, dual heaters can be used with an opposite-direction current. However, in a three-channel tundish, significant differences in flow conditions and temperature distribution between the middle channel and the side channels will occur regardless of the heating method used.

Based on previous research, this paper establishes a three-dimensional electromagnetic-flow-heat transfer coupling model. It focuses on the electromagnetic field distribution in the channels of a four-channel IH tundish when the heater rotation angles are 0°, 30°, 60°, and 90°. At the same time, the effects of electromagnetic fields on the flow and temperature distribution of molten steel are further compared. It is hoped that it will provide a new idea for ways to improve the application effect of induction-heating tundishes.

## 2. Model Description

**Figure 1:**
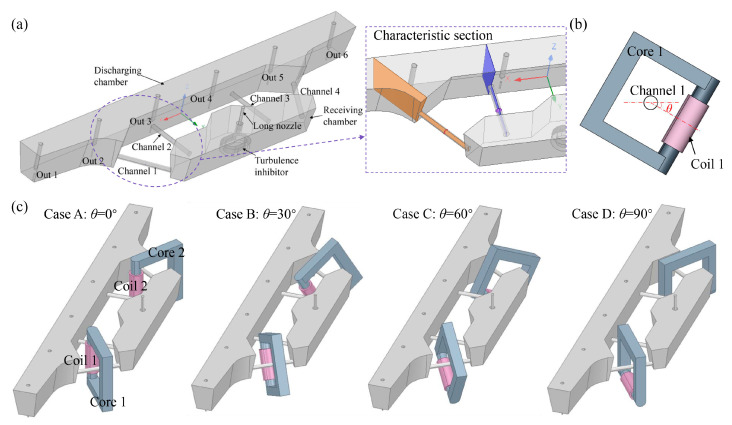
Schematic diagram of the geometric model and scheme: (**a**) schematic diagram of the structure and characteristic cross-section of the tundish body; (**b**) schematic diagram of the rotation angle of the induction heater; (**c**) schematic diagram of the relative placement of the tundish body and the heater.

The geometric structure and scheme diagram of the tundish are shown in Figure 1. The channel-type IH tundish connects the receiving chamber and discharging chamber through four channels. The coordinate origin of the model is located on the center line of each strand outlet at the bottom of the discharging chamber. Additionally, a runway-type turbulence inhibitor is set in the receiving chamber. The electromagnetic induction heating system is equipped with two heaters, symmetrically placed around channel 1 and channel 4. Therefore, in the description of the setup, induction heater 1 is used as an example. As shown in Figure 1b, the rotation angle of heater 1 around channel 1 is defined as *θ*. In Case A (*θ* = 0°), coil 1 is vertically placed between channel 1 and channel 2. In Case B (*θ* = 30°), Case C *(θ* = 60°), and Case D (*θ* = 90°), coil 1 is rotated downward by 30°, 60°, and 90° around channel 1, respectively. The schematic diagram of the relative positions of the heaters and the tundish in each case is shown in Figure 1c. Due to the symmetry of both the tundish body and the heater structure, only half of the tundish will be described in subsequent analyses. The characteristic cross-section used is shown in Figure 1a, including the longitudinal cross-sections at the location of the channels and circular cross-sections at the center of the channels. The process of establishing the electromagnetic-flow-heat coupling model for this multi-strand four-channel IH tundish has been demonstrated in the author’s previous studies [26]. In these studies, solving the electromagnetic field involves solving Maxwell’s equations, while describing the flow and heat transfer behavior of molten steel in the tundish requires solving the continuity equation, momentum equation (Navier–Stokes equation), turbulence control equation (k-ε equation), and energy equation. The computational software used includes Maxwell 2023 R1 and Fluent 2023 R1. The geometric parameters and material properties for simulation calculations are listed in Table 1. The remaining detailed information can be found in the literature [26].

## 3. Results

### 3.1. Excitation Magnetic Field Law

**Figure 2:**
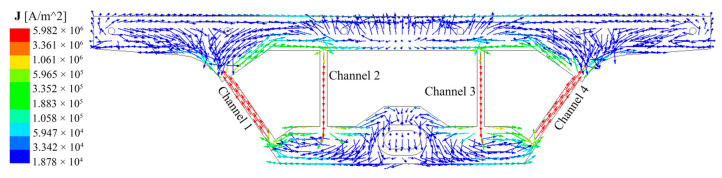
The cross-sectional current density vector diagram of the channel in Case C (*θ* = 60°).

**Figure 3:**
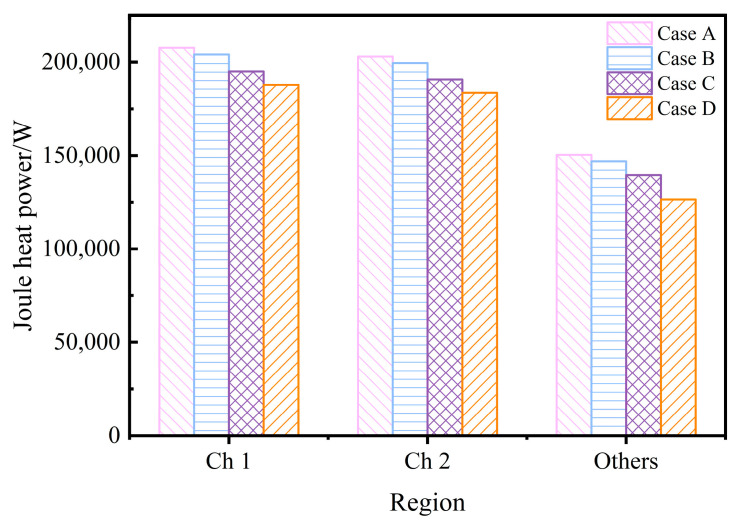
Joule heat distribution in different regions under each case.

The induction heater of the channel-type IH tundish applies an alternating current and is excited to generate an electromotive force in the molten steel. As a good conductor, molten steel can form a current loop and generate Joule heat. The Joule heat generated by the induced current directly affects the temperature of the molten steel in the tundish. Figure 2 is the cross-sectional current density vector diagram of Case C. Case C (*θ* = 60°) is taken as an example to illustrate the current loop situation in the four-channel tundish. For a four-channel IH tundish with two heaters, two closed current loops will be formed and the two loops will be symmetrically distributed. The current density in the channel is much greater than the receiving chamber and discharging chamber, so the channel is the key to heating the molten steel. Figure 3 shows the distribution of Joule heating power in different regions for each case, where the “others” include the receiving chamber and discharging chamber. The Joule heating power of each case is channel 1 > channel 2 > other regions. This is because the length of channel 1 is slightly larger than channel 2, and the molten steel area in the receiving chamber and discharging chamber is larger and the resistance is smaller. In Case A (when *θ* = 0°), the Joule heating power in each region is the highest among all cases. As the heater rotates clockwise towards the bottom of the tundish, the Joule heating power in each region gradually decreases. When *θ* is 30°, 60°, and 90°, respectively, the total Joule heating power of the tundish is 98%, 94%, and 89% of that when *θ* = 0°.

**Figure 4:**
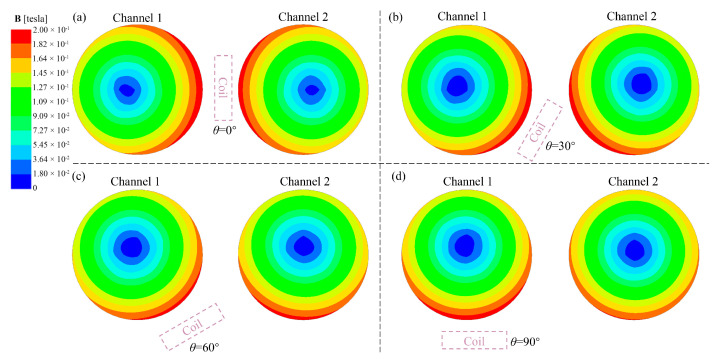
The B distribution contour of the circular cross-sections of the channels: (**a**) Case A; (**b**) Case B; (**c**) Case C; (**d**) Case D.

Figure 4 depicts the magnetic flux density (B) distribution contour of the circular cross-sections of the channels for each case. It can be observed that regardless of the angle of the heater placement, the B in the two channels is larger on the outside and smaller in the center and presents an eccentric distribution state. As the heater rotates clockwise around channel 1 towards the bottom of the tundish, the position of the maximum magnetic flux density (B_MAX_) in both channels will move downward accordingly.

**Figure 5:**
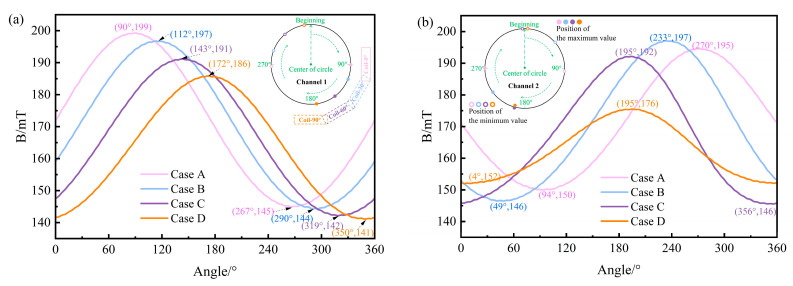
The B distribution curves of the circular cross-section characteristic lines of channels: (**a**) the circumferential lines of the channel 1 circular cross-section; (**b**) the circumferential lines of the channel 2 circular cross-section.

**Figure 6:**
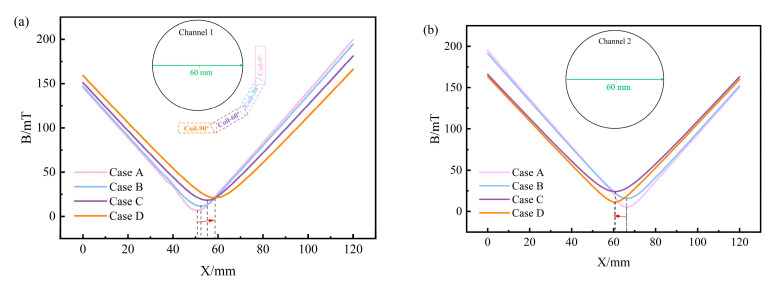
The B distribution curves of circular cross-section characteristic lines of channels: (**a**) the horizontal diameter line of the channel 1 circular cross-section; (**b**) the horizontal diameter line of the channel 2 circular cross-section.

**Figure 7:**
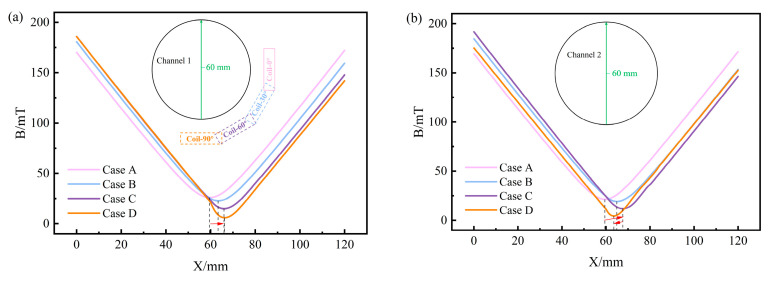
The B distribution curves of the circular cross-section characteristic lines of channels: (**a**) the vertical diameter line of the channel 1 circular cross-section; (**b**) the vertical diameter line of the channel 2 circular cross-section.

Three characteristic lines were selected on the circular cross-section of the channel for the quantitative analysis of the B distribution. Figure 5a and 5b, respectively, depict the B distribution curves along the circumferential line of the circular cross-sections for channel 1 and channel 2. The positions of B_MAX_ on the circumference line of the circular cross-section of channel 1 in Case A, Case B, Case C, and Case D are 90°, 112°, 143°, and 172°, respectively. These positions gradually move downwards as the heater rotates towards the bottom of the tundish. In addition, both the maximum and minimum values of B will gradually decrease as the heater rotates toward the bottom of the tundish. The maximum value gradually decreases from 199 mT to 186 mT, and the minimum value decreases from 145 mT to 141 mT. However, B_MAX_ on the circumference of the circular section of channel 2 in Case A, Case B, Case C, and Case D is 195, 197, 192, and 176 mT, respectively, showing a trend of increasing first and then decreasing. The minimum magnetic flux density (B_MIN_) in each case is 150, 146, 146, and 152 mT, respectively, showing a trend of decreasing first and then increasing. The extreme difference values (the difference between maximum and minimum values) are 45, 51, 46, and 24 mT, respectively. This indicates that when *θ =* 90°, the distribution of B in channel 2 is the most uniform. From the B distribution along the horizontal diameter lines of channel 1 and channel 2 circular cross-sections in Figure 6a,b, it can be observed that as the heater rotates downward around channel 1 in the clockwise direction to *θ =* 90°, the position of B_MIN_ along the horizontal diameter lines of both channel 1 and channel 2 is closest to the center, indicating a lighter horizontal eccentricity condition. Furthermore, examining the B distribution along the vertical diameter lines of channel 1 and channel 2 circular cross-sections in Figure 7a,b, it can be noted that as the heater rotates towards the bottom of the tundish, the position of B_MIN_ along the vertical diameter lines of the channel 1 circular cross-section gradually moves away from the center. Conversely, the distance between the position of B_MIN_ along the vertical diameter lines of the channel 2 circular cross-section and the center first increases and then decreases, reaching a peak at *θ =* 60°.

**Figure 8:**
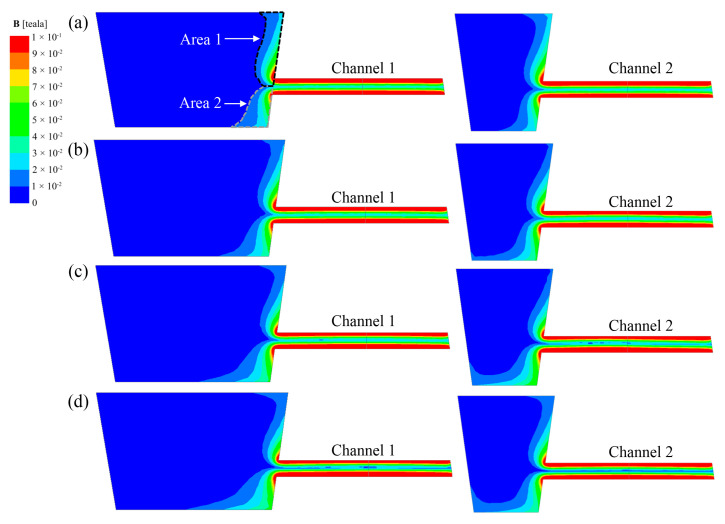
The contour diagram of B distribution in the longitudinal section of the channel location under each case: (**a**) Case A; (**b**) Case B; (**c**) Case C; (**d**) Case D.

**Figure 9:**
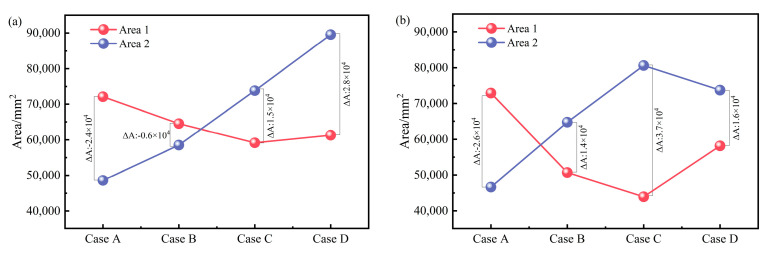
Comparison of the effective area of the longitudinal section B contour diagram at the location of the channel: (**a**) channel 1; (**b**) channel 2.

Figure 8 shows the contour diagram of B distribution along the longitudinal cross-sections of channels for each case. It can be seen from the figure that regardless of the relative placement angle between the heater and the channel, the B distribution in the two channels is that the edges are significantly higher than the center of the channel. Moreover, the B value near the tundish wall is greater in the discharging chamber, while it decreases further away from the wall. The above phenomena are all manifestations of the skin effect of the magnetic field. It is considered that B exceeding 0.01 T is the effective magnetic field area [27]. Taking channel 1 of Case A as an example, the effective B area of this section in the discharging chamber is shown in Figure 8a. The boundary at Z = 300 mm of the channel center in the vertical direction is defined as the boundary between the upper and lower effective B areas, denoted as Area 1 and Area 2, respectively. The numerical statistics of Area 1 and Area 2 for each case are shown in Figure 9, where ΔA_B_ = Area 2 − Area 1. The ΔA_B_ of channel 1 is negative in Case A and Case B and positive in Case C and Case D, and ΔA_B_ gradually increases as the coil rotates toward the bottom of the tundish. The ΔA_B_ of channel 2 is negative only in Case A and positive in other schemes, and it has a maximum value in Case C. According to the formula for EMF calculation, EMF is directly proportional to B. A larger absolute value of ΔA_B_ indicates a greater difference in electromagnetic force in the vertical direction (F_Z_), making it easier for the flow at the channel outlet to exhibit an upward or downward trend.

**Figure 10:**
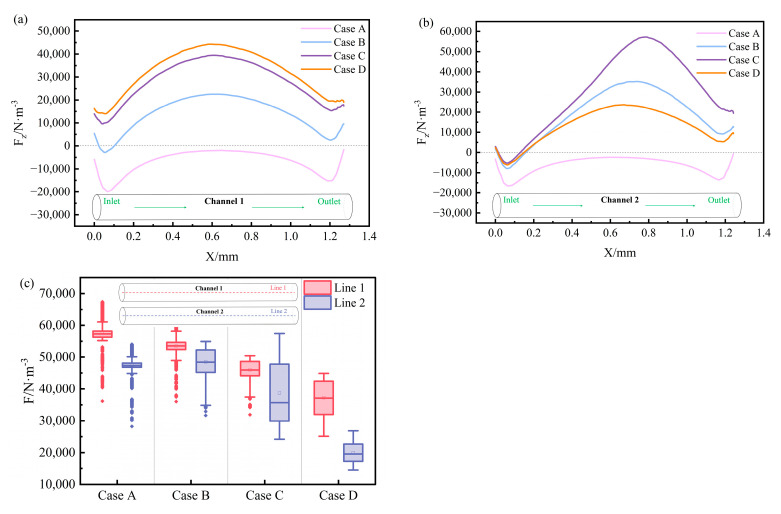
Distribution of EMF at the centerline of each channel: (**a**) F_Z_ distribution curve at the centerline of channel 1; (**b**) F_Z_ distribution curve at the centerline of channel 2; (**c**) EMF at the centerline of the two channels.

Figure 10 shows the EMF distribution at the center line of the channel under each scheme. Figure 10a,b are the F_Z_ distribution curves of the center lines of channel 1 and channel 2. The positive and negative values of F_Z_ in the figure represent the direction. The positive value means the F_Z_ value is upward, and the negative value means the F_Z_ value is downward. From the figure, it is evident that the EMFs along the centerlines of channel 1 and channel 2 for Case A are both directed downward. The F_Z_ distribution of the two channels is a ‘W’ shape. The F_Z_ value changes significantly near both ends of the channel (inlet and outlet), while the F_Z_ value in the middle of the channel approaches 0. The F_Z_ values of the center lines of channel 1 and channel 2 in Case B, Case C, and Case D are almost all directed upward, and they all have a distribution trend of being large in the middle and small at the two ends. The heater gradually rotates around channel 1 toward the bottom of the tundish, and the upward F_Z_ of the center line of channel 1 gradually increases. Furthermore, when *θ =* 60°, the upward F_Z_ along the centerline of channel 2 is the largest, which aligns with the previously mentioned pattern of ΔA along the longitudinal cross-section of the channel. Figure 10c is a box plot of the electromagnetic resultant force value at the center line of the channel. As the heater rotates gradually towards the bottom of the tundish around channel 1, the EMF along the centerline of both channels shows a decreasing trend. Especially when *θ* rotates from 60° to 90°, the EMF at the centerline of channel 2 decreases significantly. It shows that *θ =* 90° will significantly reduce the EMF exerted on the molten steel.

### 3.2. Electromagnetic Field Effects

**Figure 11:**
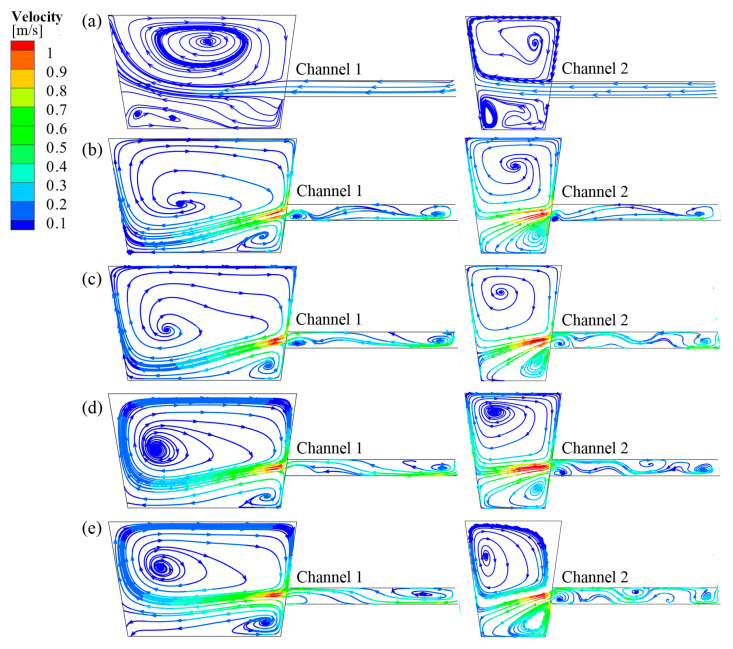
Streamline diagram of the longitudinal cross-sectional of channel for each case: (**a**) without IH; (**b**) Case A; (**c**) Case B; (**d**) Case C; (**e**) Case D.

Figure 11 depicts streamline diagrams of longitudinal cross-sections at the channel positions for each case. As the heater gradually rotates towards the bottom of the tundish around channel 1, the outflow of molten steel from channel 1 changes from downward flow to horizontal flow. When *θ* is 60° and 90°, the outflow of molten steel from channel 1 no longer washes the bottom of the tundish. Additionally, when *θ =* 60°, the outflow of molten steel from channel 2 approaches horizontal flow the most, whereas when *θ =* 90°, the downward flow trend of the outflow from channel 2 intensifies, which is consistent with the distribution of B and EMF along the longitudinal cross-sections at the channel positions. Comprehensively comparing the outflow of molten steel from channel 1 and channel 2, it can be concluded that Case C (when *θ =* 60°) is more conducive to preventing erosion at the bottom of the discharging chamber.

**Figure 12:**
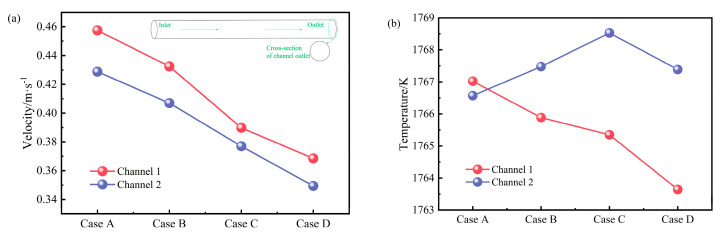
The average flow velocity and average temperature of the circular cross-section molten steel at the channel outlet: (**a**) velocity; (**b**) temperature.

Figure 12 shows statistics of the average flow velocity and average temperature of molten steel with a circular cross-section at the channel outlet. It can be seen from the figure that regardless of the placement angle of the heater, the flow velocity of the molten steel outflowing from channel 2 is lower than that of channel 1. As the heater rotates around channel 1 toward the bottom of the tundish, the exit velocities of both channels gradually decrease. The outlet flow velocity of channel 1 drops from 0.46 m s^−1^ to 0.37 m s^−1^, and the outlet flow velocity of channel 2 drops from 0.43 m s^−1^ to 0.35 m s^−1^. As the heater rotates towards the bottom of the tundish around channel 1, the outlet temperature of channel 1 gradually decreases from 1967.02 K to 1763.64 K, while the outlet temperature of channel 2 initially increases and then decreases, reaching a maximum value of 1768.53 K when θ = 60°. This shows that the solution with a heater rotation angle of 60° can reduce channel erosion to a certain extent and can also more effectively exert the heating effect of the induction heater on the molten steel inside the channel.

## 4. Conclusions

This study extensively analyzed the influence of different heater placement angles on the electromagnetic field distribution in a four-channel induction-heated tundish through numerical simulation. It proposed using the channel centerline as a boundary and the difference in effective magnetic flux density area (ΔA_B_) between the upper and lower parts of the channel as a quantitative evaluation criterion to analyze the magnetic flux density (B) distribution state along the longitudinal cross-section of the channel. Furthermore, it elucidated the effect of electromagnetic field results on the flow of molten steel and temperature distribution. The main conclusions are as follows:

1. Regardless of the relative placement angle between the heater and the channel, the distribution of B at the center circular cross-section of the channels is consistently eccentric. As the heater rotates towards the bottom of the tundish around channel 1, the distribution of B at the center circular cross-section of the channels transitions from a horizontal eccentricity to a vertical eccentricity.
2. As the heater rotates towards the bottom of the tundish around channel 1, the ΔA_B_ of channel 1 gradually increases from −2.4 × 10^4^ mm^2^ to 2.8 × 10^4^ mm^2^, while the ΔA_B_ of channel 2 has a maximum value of 3.7 × 10^4^ mm^2^ at *θ* = 60°. At the same time, the distribution pattern of the vertical electromagnetic force along the centerline of the channels aligns with the pattern of ΔA_B_.
3. Compared to the conventional placement of the heater, when the heater rotation angle is 60°, the channel 1 and channel 2 outlet cross-sectional flow velocities are decreased by 15% and 12%, respectively, while the channel 2 outlet temperature is increased by 1.96 K. The molten steel outflow from channel 2 no longer exhibits significant downward flow, which is more conducive to reducing scouring at the bottom of the tundish channels and discharging chamber. At the same time, it enables a more effective utilization of the induction heater for heating the molten steel inside the channels.
4. This study did not consider the spatial constraints of the heaters and the tundish body in practical applications. Installation issues will be addressed in actual use, and further design of the heater’s geometry will be undertaken to fit the available space on-site.

## Figures and Tables

**Table 1:** Geometric structure parameters and physical property parameter table.

Parameters	Values	Parameters	Values
Height of channel	0.3 m	Relative permeability of each material	1
Diameters of channel	0.12 m	Conductivity of molten steel	7.14 × 10^5^ S/m
Distance between the strands	1.9 m	Viscosity of molten steel	0.0055 kg/(m·s)
Melting depth in discharging chamber	0.85 m	Density of molten steel	8237–0.827 T
Length of channel 1	1.27 m	Thermal conductivity of molten steel	35 W/(m·K)
Length of channel 2	1.24 m	Specific heat of molten steel	824 J/(kg·K)
Ampere-turn numbers	3.66 × 10^4^	Inlet velocity	1.19 m/s

T is temperature.

## Data Availability

The original contributions presented in the study are included in the article, further inquiries can be directed to the corresponding authors.
